# Editorial: Advancing Methods for Psychological Assessment Across Borders

**DOI:** 10.3389/fpsyg.2019.00503

**Published:** 2019-03-19

**Authors:** Kai Ruggeri, Lana Bojanić, Lindsey van Bokhorst, Hannes Jarke, Silvana Mareva, Olatz Ojinaga-Alfageme, David T. Mellor, Sam Norton

**Affiliations:** ^1^Department of Health Policy and Management, Columbia University, New York City, NY, United States; ^2^Centre for Business Research, Judge Business School, University of Cambridge, Cambridge, United Kingdom; ^3^Centre for Mental Health and Safety, University of Manchester, Manchester, United Kingdom; ^4^Faculty of Psychology and Neuroscience, Maastricht University, Maastricht, Netherlands; ^5^RAND Europe, Cambridge, United Kingdom; ^6^MRC Cognition and Brain Sciences Unit, University of Cambridge, Cambridge, United Kingdom; ^7^Department of Psychological Sciences, Birkbeck College, University of London, London, United Kingdom; ^8^Center for Open Science, Charlottesville, VA, United States; ^9^Psychology Department, Institute of Psychiatry, King's College London, London, United Kingdom

**Keywords:** psychological methods, reproducibility, multinational research, replication, early career researchers

## A New Generation of Psychological and Behavioral Scientists

The nascent push for greater transparency and reproducibility in psychological and behavioral sciences has created a clear call for better standards for research methods across borders and languages. Exponential growth in computing power, access to secondary data, and widespread interest in new statistical methods have ensured a new generation of behavioral researchers will have opportunities for discovery and practice on a level without precedent. As of publication, more than 200 institutions in over 100 countries globally have launched behavioral policy units, and there is immeasurable interest in applications from across specialty areas in psychology. With these trends, there is an undeniable and growing demand for improved methods for assessment in scientific study, industry, and policy.

To maintain progress, it is critical that the next generation of researchers have the awareness, training, and practice for conducting high-quality research in psychological assessment, particularly when studying across populations, borders, and languages. This Editorial summarizes key insights for the Research Topic *Advancing methods for psychological assessment across borders*, followed by general guidance for early career researchers working in multiple languages and countries, or when adapting existing methods for new settings and populations.

## Edition Insights

This Research Topic was launched to support professional development of students and early career behavioral scientists in the Junior Researcher Programme, an initiative that supports six multi-country psychological research projects annually. Senior academics were also invited to contribute manuscripts of their own multinational studies. Mirroring the field generally, there is considerable diversity in the 19 published manuscripts, with protocols and early-stage findings in education, health, development, technology, personality, data privacy, social media, organizational leadership, and financial decision-making.

The first wave of papers covered a variety of techniques for testing existing paradigms with greater cross-cultural appreciation. This included the development of a multilingual app for assessing quality of life, an expanded measurement for standardizing cognitive ability scores across Europe, new approaches to personality measures to link with cognitive ability, measurement of how teachers support students emotionally, psychological constructs and accuracy of subjective scoring in gymnastic judges, and a cross-validation of an empathy scale. Following topics covered new approaches in assessment across mental health and decision-making. Papers included learning methods, the dark triad of personality, moral behavior, and psychopathy from a neuroscience communication lens, the impact of music on the well-being of elderly people, adolescent well-being, validation of PHQ-9 in Norwegian, and assessing videos as an intervention tool on social media. In the final wave, the focus shifted toward more narrow assessment of behaviors, such as the influence of social norms on eating behavior during pregnancy, parental decision to have children vaccinated, Facebook use and preference for privacy options, how identity leadership builds organizational commitment, and the decision to donate to charity. Such diversity in papers highlights the need for early career researchers to have robust training and experience in responsible, replicable scientific methods.

This Research Topic is primarily geared toward Protocols, meaning there is less in the way of new evidence to summarize. Instead, we cogitate on the approaches, challenges, reviewer feedback, and general direction from these manuscripts as a means of guiding the next wave of junior researchers.

## Guidance for Early Career Researchers

Across these Protocols, a number of themes emerge in attempts to conduct psychological research across borders, for instance:

1. Translation into a new language is not the only adaptation existing measurements need to function in new settings—there is no one clear answer on how best to conduct comparisons in all cases (i.e., using the items that work well enough in all settings vs. using the items that best fit each location);
2. When designing studies to be carried out in multiple countries, particularly with limited resources to conduct the study, it may be more useful to focus on common, narrowly-defined groups in each, than attempt to be representative across all populations for all countries tested
3. Hungry for data: while positive in terms of how it reflects the enthusiasm of the next generation of behavioral scientists, the omnipresent wish to add more items, recruit more participants, and do more rounds of collection may serve as a barrier to producing high-powered studies on clear questions;
4. Highly cited papers that appear in textbooks over several generations of students may have a sample size (power) that would not even pass as a pilot study under present research standards;
5. WEIRD (Western, educated, industrialized, rich, and democratic) populations are easier to access; studying non-WEIRD populations may offer substantially more insight than simply improving existing instruments in tested populations;
6. Particularly when using extensive assessments, knowing how to position a study for eventual publication is a major challenge, and can slow the development of new insights, particularly when a key feature is the adaptation into multiple languages. This is particularly relevant for junior researchers (see Table 1).

**Table 1:** List of common study designs for early career researchers.

	**Features**	**Considerations**
Proof of concept	New method or intervention No strong outcome hypothesized Emphasis on practical delivery of study, not insights	An ideal way to simply assess if a given approach is useful from a study perspective, but typically harder to publish unless truly novel and useful
Pilot study	A small-scale trial of method or modality Emphasis on ability to elicit effect rather than on size, strength, or direction of effect Insights reported but not interpreted	Approach to be used when all the necessary features are present for a major scientific study but concerns about practical delivery give reason to withhold a full wave of data collection that has a clear risk of a systematic flaw
Feasibility study	Research to test study practicality, materials, management, outcomes Answers the question “Can this study be done?” Emphasis on the feasibility of the study design, rather than on effects Insights only reported on process	Methods that are used in a feasibility study range from interviews to forecasting. Commonly overlooked aspects include budget planning and attitudes of potential participants toward the topic of research; latter is crucial in multicultural research.
Exploratory study	Clear theoretical framework for the primary question No strong hypothesis or prediction about patterns or specific outcomes/interactions, but broad reporting of insights	Preferable when the literature and research team have strong basis for no clear hypothesis but can create scenario for cherry-picking or overwrought methods/conclusions
Original confirmation	Full study with strong theoretical justification Specific hypotheses and prediction	When there is a clear theoretical argument that benefits from further testing, but a modified approach is preferable to direct replication. Especially useful when seeking convergence on a topic, rather than explicit reproduction.
Replication	A study that aims to replicate the findings of another study on another population or in a different setting.	Precise operationalization is the key. Only when the concept of interest is precisely defined, the success or the failure to replicate may be attributed to the (non)existence of the effect.
Validation of a metric	Measures the ability of a new/adapted instrument to capture a phenomenon. Emphasis on discriminant features between relevant individuals and groups, and possible change.	Validation is likely to be an iterative process, involving collaborative thinking, and a various number of pilot studies. It is wise to always keep face-validity in mind.
Revalidation of metric in new setting or language	Investigation to check if an instrument measures the same construct in different populations	Useful if no measure for a construct exists in a specific setting or if there are reasons to assume a metric does not equally apply to specific circumstances

## Recommendations

While difficult to provide uniform guidance for all international, multilingual, and other multi-site studies in psychological sciences, there are some common steps that can be taken to ensure a smoother process with greater possibility of meaningful insights, while avoiding common pitfalls. We highlight some of these here:

### Focus the Content on What Matters

Empirical papers usually revolve around a primary research question. All text written in the build up to the research question should serve the reader to understand why this question is important. Although researchers often wish to be as thorough and detailed as possible, too much information creates confusion and detracts from a primary message. Be concise and stay on point. Unless absolutely necessary and directly relevant, do not go back to Freud and Jung, and remove tangents or overstated contingencies. Focus on the critical assumptions and make sure no reader has to guess what the question or hypothesis are.

### Utilize the Open Science Framework

At the time of writing, the mandate for researchers to ensure transparency and reproducibility in research is young but building. To meet these standards, there are a broad range of resources, tools, guidelines, and examples produced by the Open Science Framework (osf.io). Early career researchers are encouraged to manage project materials, such as questionnaires, instructions, analysis scripts, and datasets in an OSF project (See Supplement 1).

### Pre-register Studies

Pre-registration has been suggested as an important tool to combat publication bias and questionable research practices and improve the transparency of the research process (Munafò et al., 2017). Pre-registrations are time-stamped documents specifying all plans for methods, data collection, and analysis. These are produced prior to conducting study. Such documents are expected to settle crucial decisions of the research process *a priori*, along with transparency about initial hypotheses (Nosek et al., 2018). There may be instances where simulated or pilot data may be necessary to aid in certain aspects of methodological decision-making, but this should be justified if done. Another alternative is to randomly split datasets in order to conduct exploratory analyses prior to confirmation with the held off, un-analyzed dataset (Anderson and Magruder, 2017; Supplement 2).

Pre-registrations must eventually be made public in order to address underreporting biases, but many may be embargoed for various lengths of time (e.g., up to 4 years on the OSF^1^) be private or public. They also may be submitted for peer review as Registered Reports^2^ prior to data collection, such that valuable feedback could be obtained ahead of the project execution when early career researchers would be most likely to benefit from it. Researchers should also adhere to institutional review board (IRB) guidelines and include relevant approvals and ethical guidance when they pre-register.

### Replicate Before You Explore

Registered reports reviewed and accepted prior to data collection provide incentives for researchers to conduct valuable replication studies. Ambitious early career researchers may initially be more drawn to the thought of testing a completely new idea of their own. However, in attempting to build new hypotheses based on theories that may not have substantial validation, the researcher may find themselves with a lot of unpublishable material. Instead, consider first replicating a critical finding that produces the assumptions for your own work, and see if it holds. If it does, then finding a new avenue to explore can be possible. If it does not, then you have ample opportunity to discover unexpected moderating variables. Either way, you have now made an important contribution to the field while concurrently allowing yourself both confirmatory and exploratory hypotheses to test. Using the Registered Report model for this first step can be crucial, as “successful” replications are often deemed “too boring” to publish, whereas “unsuccessful” replications may be subject to more intense scrutiny than warranted and face obstacles to publication.

### Publish Null Findings

We optimize the possibility of finding an effect through power calculations to inform sample sizes, but sometimes our results turn out to be null. Albeit often less desirable, such findings are equally relevant and should not be overlooked by researchers or publishers. Data syntheses and systematic reviews rely on publications of statistically significant effects as well as null findings to yield an accurate and generalized conclusion. Not publishing null findings therefore results in a skewed representation of the reality. Null findings could also inform future studies by providing context to consider potential confounds and moderators. So no matter if it is reject or fail to reject, start with publication in mind.

### Apply for Ethical Approval Early

All researchers should seek guidelines for obtaining necessary ethical or IRB clearance as early as possible. As a minimum, review should be obtained at the institution of the principal investigator. When testing in multiple settings, approval may need to be sought from additional boards, such as schools (for collecting student data) or organizations (for collecting data on a centralized platform). Ethical reviews will usually include comments on methods and legal documents such as privacy notices, so earlier review would be better for finalizing research plans, particularly pre-registration. No two IRBs are guaranteed to function in the same way, but most will focus more on protecting participants in the study and the institution, and less on getting you a high-impact publication. We advise you consider methodological input from trusted experts before and after ethical review to ensure the highest quality study. Finally, try to consider aspects of data sharing (Meyer, 2018) and overall project transparency in your IRB applications in order to not face hurdles to transparency later on (Supplement 3).

### Use the Oxford Comma

Please.

## Challenges

Put bluntly: the goalposts have changed in research. While concerns about replication and sufficiently-powered studies have raised standards across the behavioral sciences, they have also created unprecedented challenges for early career researchers. For example, classic rules of thumb for testing new surveys are no longer permissible and should be replaced with systematic power calculations preceding data collection. While this will unambiguously improve scientific quality, it also requires both the statistical knowledge to produce those estimates as well as the resources to meet those participation thresholds. Likewise, while we are fully in support of pre-registering studies, journals note the difficulty in finding reviewers willing to engage with these, which can slow down the completion of study on time-limited research projects conducted by students.

Current standards developed over time and were not used by previous generations of researchers, meaning new behavioral scientists are being trained by academics who were not subject to them at the same stage in their careers. Even when these new approaches become standard in university lectures, alternative learning resources, hands-on experience with research, and peer-learning are crucial for the development of junior researchers.

## Conclusions

Take every word here as a positive. With new challenges come new opportunities, and the next wave of behavioral researchers will have a tremendous impact on society. The earlier that the new standards in the field can be applied across all studies, the (likely) better this will be for the advancement of the field and public perception of the work. As psychological and behavioral scientists cover all domains of life for individuals and societies, this will surely promote the greatest impact for the well-being of the science and of populations. Your professional ancestors are cheering for you.

## Author Contributions

KR directed, drafted, framed, edited, proofed, and finalized the entire paper. All other authors contributed equally by proofing, editing, and making direct contributions to various sections of the text.

### Conflict of Interest Statement

DM is an employee of the Center for Open Science, which builds and maintains the free and open source platform, OSF. The remaining authors declare that the research was conducted in the absence of any commercial or financial relationships that could be construed as a potential conflict of interest. The handling editor is currently editing co-organizing a Research Topic with the author KR, and confirms the absence of any other collaboration.

## Footnotes

^1^*http://help.osf.io/m/registrations/l/524205-register-your-project#Choose-your-privacy-settings*

^2^https://cos.io/rr
