# Supplementary material for: Editorial: Advancing Methods for Psychological Assessment Across Borders
Source: Front Psychol. 2019 Mar 19;10:503. doi: 10.3389/fpsyg.2019.00503 (PMC6436195; doi:10.3389/fpsyg.2019.00503)
Supplement: Supplementary file 1 [file Table_1.DOCX]

**Supplement: Involving peer reviewers and stakeholders in the design of empirical study**

Peer reviewers are commonly involved in improving the quality of an academic paper. However, designing a research study is an iterative process where peer reviewers can be extremely helpful in refining the study design before the study commences. For example, students can consult their academic supervisors, while early career researchers may have access to a departmental mentor network or can seek advice from senior academics. The input from peer reviewers can provide guidance towards a more appropriate methodology otherwise not considered, and having someone review with a fresh look may expose methodological flaws or inconsistencies. Don’t have anyone in-house to advise? Try Academic Twitter. A formalized method for this process is through the Registered Reports workflow, though another somewhat formal process could be submitting a proposed study design to a dedication protocols journal or simply to a preprint serve to help solicit input for improvement.

Each study defines a specific target group as study participants. Stakeholder involvement should not be treated with a ‘tick box’ mentality in order to meet ethical or other requirements. Any study that provides materials to participants, such as consent forms and information sheets, will benefit from engaging stakeholders who can reveal issues with readability, academic jargon, and general understandability of a text which researchers may overlook. Stakeholders can also provide insights on study practicality, integration, and acceptability.

**1**

A comprehensive guide to starting projects, sharing materials, and managing collaborations is available at <http://help.osf.io/>, including best practices in organizing files, creating a data management plan, and a data dictionary to ensure that variable naming conventions are interpretable by future readers (including yourself!): <http://help.osf.io/m/bestpractices>.

2

See the Center for Open Science informational page for more examples, templates, references, and FAQs regarding pre-registration: https://cos.io/prereg

3

See https://osf.io/g4jfv/ for example language for IRB applications and informed consent templates and Meyer (2018) for more practical tips in this process.
